# Exploring the dynamics of sports records evolution through the gembris prediction model and network relevance analysis

**DOI:** 10.1371/journal.pone.0307796

**Published:** 2024-09-19

**Authors:** Lu Tang, Mingliang Yang

**Affiliations:** 1 Department of Physical Education, Civil Aviation Flight University of China, Guanghan, China; 2 Institute of aviation sports, Civil Aviation Flight University of China, Guanghan, China; Manipal Academy of Higher Education, INDIA

## Abstract

**Background:**

Sports records hold valuable insights into human physiological limits. However, presently, there is a lack of integration and evolutionary patterns in the recorded information across various sports.

**Methods:**

We selected sports records from 1992 to 2018, covering 24 events in men’s track, field, and swimming. The Gembris prediction model calculated performance randomness, and Pearson correlation analysis assessed network relevance between projects. Quantitative study of model parameters revealed the impact of various world records’ change range, predicted value, and network correlation on evolutionary patterns.

**Results:**

1) The evolution range indicates that swimming events generally have a larger annual world record variation than track and field events; 2) Gembris’s predictions show that sprint, marathon, and swimming records outperform their predicted values annually; 3) Network relevance analysis reveals highly significant correlations between all swimming events and sprints, as well as significant correlations between marathon and all swimming events.

**Conclusion:**

Sports record evolution is closely linked not only to specific sports technology but also to energy expenditure. Strengthening basic physical training is recommended to enhance sports performance.

## Introduction

In competitive sports, achieving a new world record is always gratifying. However, it’s not just about representing the pinnacle of a specific sports specialty, it’s crucial to assess the valuable information and patterns behind the record [1]. Sports records, over time, follow a monotonic function on a time scale and spatial function [2]. Hence, every sports records is bound to be surpassed eventually. Factors like gender, age, genetics, participant numbers, geopolitics, doping, technological prowess, and athletic training contribute to creating sports records, with athletic training playing a decisive role [1, 2].

Sports training serves as a synthesis of research outcomes from diverse disciplines to enhance athletic performance and applies the laws and characteristics of one sport to training in others. It advances sports science by integrating insights from mathematics, physics, computational science, engineering, anatomy, and physiology. Simultaneously, it extrapolates laws and features from training theories like item clusters, integration, parallelism, crossover, and multimodality, guiding training practices [3–6], and offering a theoretical foundation for selecting athletes across sports.

The essence of sports lies in the amalgamation of external movements and internal bodily functions. The variability in function among different sports is a crucial factor influencing the existence of laws between them. Sports records, epitomizing the pinnacle of human body movement function for a given year, not only reflect the highest sports training standards but also encapsulate the features and laws governing diverse disciplines. Consequently, this study aims to correlate and analyze 24 sports records from 1992 to 2018, exploring the interaction laws between world records and the evolving human function in each sport. This endeavor enriches quantitative research in sports training science and exercise physiology.

## Methods

### Data source

Data were sourced from the International Amateur Athletic Federation (IAAF) (www.worldathletics.org) and the International Swimming Federation (Fédération Internationale de Natation Association, FINA) (www.fina.org). Considering the prevalent use of banned substances to enhance athletic performance in the 1970s and 1980s, this study selected the annual world records of 648 male athletes from 1992 to 2018 in track (100m sprint, 200m sprint, 400m running, 800m running, 3000m running, 10,000m running, 400m hurdles, and marathon), field (high jump, long jump, and triple jump), and swimming (100m backstroke, 200m backstroke, 100m breaststroke, 200m breaststroke, 100m butterfly, 200m butterfly, 50m freestyle, 100m freestyle, 200m freestyle, 400m freestyle, 800m freestyle, 200m medley, and 400m medley). These events span competitions like the Olympic Games, the World Championships in Athletics, and the World Swimming Championships.

### Statistical analysis

#### Gembris predictive model

Correlations between data fluctuations would be meaningless if random factors caused fluctuations in sports world records. To explore the presence of randomness in these fluctuations, the impact of random factors on world record fluctuations was evaluated using Gembris predictive statistical model [1, 7]. The annual world record fluctuation is presumed to be a smooth stochastic process devoid of systematic progression and adheres to a Gaussian distribution (mean *μ* and standard deviation *σ*). The anticipated value of the record over the next *N* years can be estimated as:

$$x_{max}=\mu +\sigma {\left(a_{0}+a_{1}\text{ln}\;\text{ln}\left(N\right)\right)}^{2}$$ (1)

where the optimal coefficients are *a*_*0*_ = 0.818, *a*_*1*_ = 0.574, and *a*_*2*_ = 0.349. These coefficients, along with *σ* = 1, ensure that the error in approximating xmax remains below 0.06 within a specified interval. Similarly, the standard deviation of the approximation of *x*_*max*_ is:

$$\sigma_{E}=\sigma (b_{0}+b_{1}{\text{ln}\;\text{ln}\;N+b_{2}(\text{ln}\;\text{ln}\;N))}^{2}$$ (2)

The optimal parameters, *b*_*0*_ = 0.8023, *b*_*1*_ = -0.2751, and *b*_*2*_ = 0.0020, result in an approximation error of under 0.15 percent. This paper utilizes data from 1992–2001 to project trends in world records from 1992–2018, and the estimated fluctuations are subsequently compared with actual sports performance results.

#### Network relevance

The records were documented as relative fluctuations by taking the 1992 world records as the baseline for each subsequent year’s records. The division of the world records of other years by the baseline determined the relative fluctuations. Using SPSS 22.0 software (IBM, NY, USA), we calculated the Pearson correlation coefficients for the fluctuation patterns of each world record, setting the significance level for statistical analysis at *P* = 0.05. The time series of relative fluctuation (TSRF) for each world record x can be expressed as follows:

$${TSRF}_{x}\left(j\right)=\frac{x_{j}}{x_{i}},(i=1992,\;j=1993,1994,\ldots ,2018)$$ (3)

The number of interrelationships between any two TSRFs reflects the temporal correlation of the evolution of world records. Given that speed events and jumping events set world records based on the minimum and maximum values, respectively, this paper transforms the world records of jumping events inversely to facilitate a direct comparison with the former. The correlation coefficient (*R*) is employed to assess the strength of correlation in the fluctuation of world records between events. A value of *R* closer to 1 indicates a stronger positive correlation.

## Results

### Evolution of sports records

**Fig 1:**
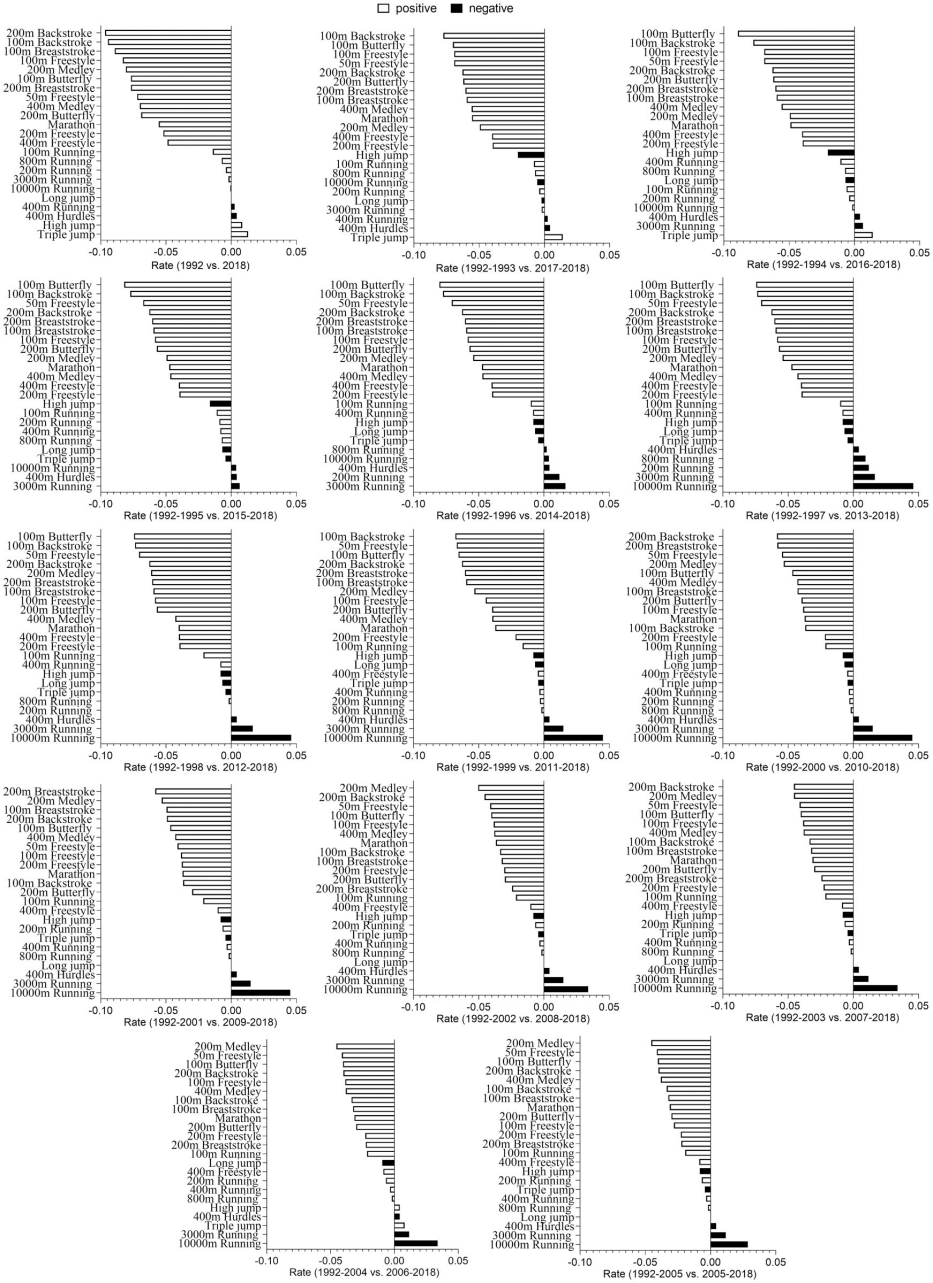
Magnitude of change in sports records. White columns show growth in sports records; black columns show negative growth in sports records.

To avoid errors caused by random fluctuations in data, we selected the best world records for each year from 1992 to 2005 to compare with the records for each year from 2005 to 2018. Fig 1 provides a general overview, indicating that swimming events generally exhibit a greater magnitude of change than track and field events. Notably, the 200m backstroke world record saw a remarkable 10% improvement in 2018 compared to 1992. Significance is the marathon, the only track and field event surpassing certain swimming events in improvement over the years, with a direct 5% enhancement in 2018 over the 1992 record. The marathon ranks 7th among all events in the spans of 1992–2002 and 2018–2008. Except for the marathon, other track and field events experienced a substantial decrease in change magnitude compared to swimming records. Specifically, the 400m freestyle, with the smallest increase in 2018 compared to 1992, was 3.46 times higher than the 100m sprint, which had the largest increase in track and field events. Furthermore, high jump, long jump, 400m hurdles, 200m running, 400m running, 800m running, 3,000m running, and 10,000m running all exhibited negative growth in all year span comparisons (Fig 1).

### Prediction of sports records

**Fig 2:**
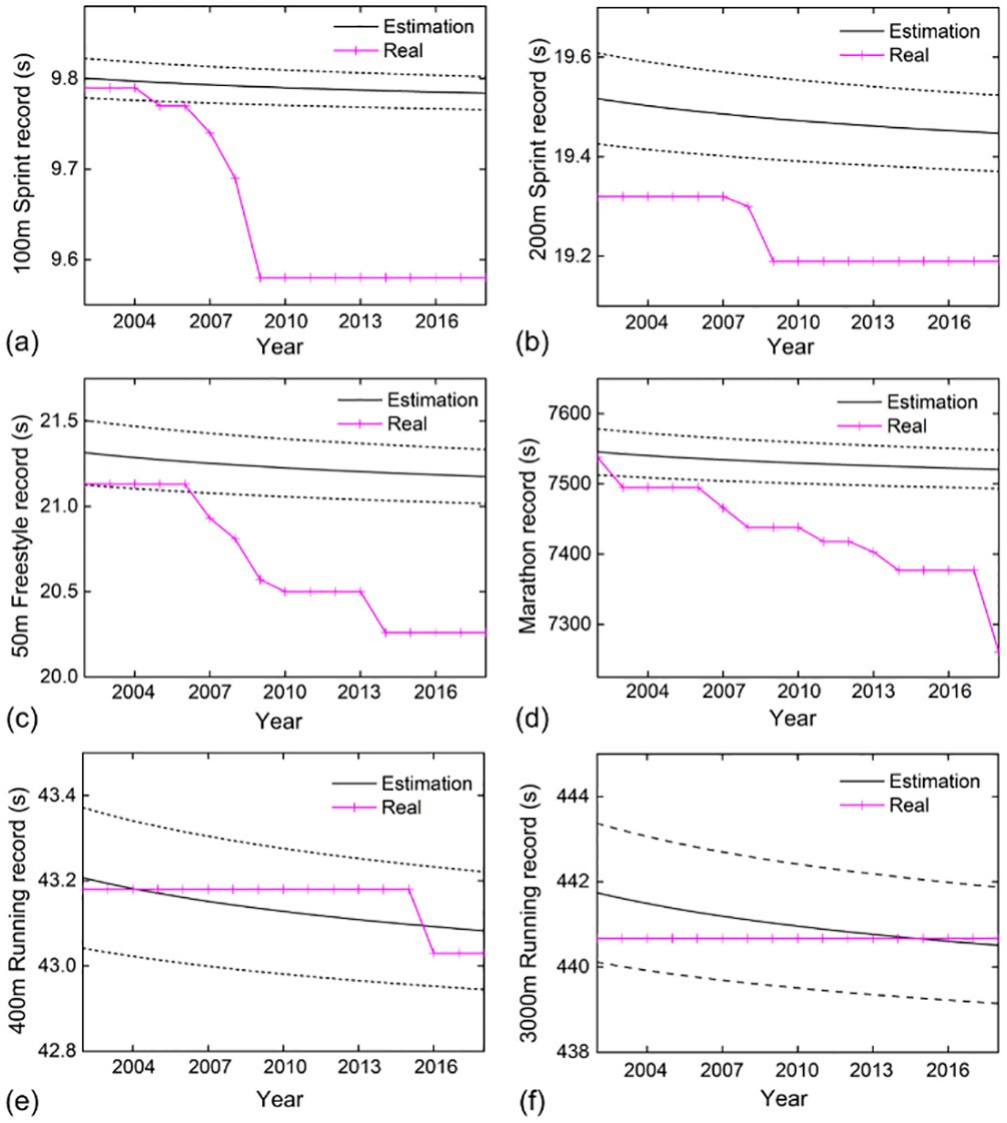
Gembris predicts the evolution of sports records between 2002–2018. **(a)** 100m sprint; **(b)** 200m sprint; **(c)** 50m freestyle; **(d)** marathon; **(e)** 400m running; **(f)** 3000m running. The black solid line and the red solid line indicate the theoretical estimate and the actual value, respectively, and the dashed line indicates the data change *σ*_*E*_ interval.

The prediction results indicate that except for middle and long-distance running, track and field events fall within the *σ*_*E*_ interval (Fig 2). However, all statistics related to sprinting, marathon, and swimming events deviate from the *σ*_*E*_ interval. Fig 2e and 2f reveal that the stochastic variation process of the world records for 400m and 3000m running aligns with the actual performance evolution. In contrast, the 100m sprint, 200m sprint, 50m freestyle, and marathon events exhibit significant deviations (Fig 2a and 2d).

### Network correlations in the evolution of sports records

**Fig 3:**
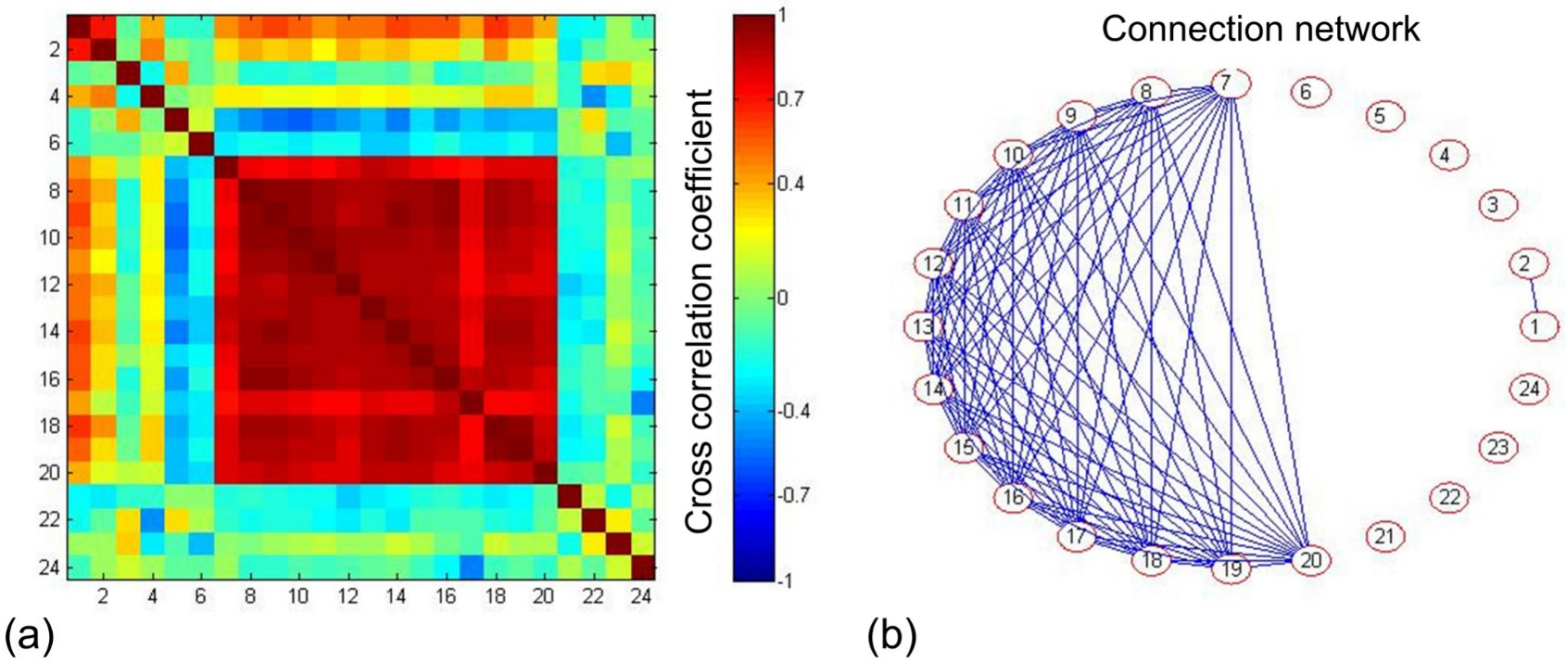
Network relevance of sports records. (a) Matrix of correlation coefficients; (b) Connected network of 24 sports records evolved, two TSRFs with correlation coefficients greater than 0.7 are defined as connected. (1, 100m sprint; 2, 200m sprint; 3, 400m running; 4, 800m running; 5, 3000m running; 6, 10,000m running; 7, marathon; 8, 100m backstroke; 9, 200m backstroke; 10, 100m breaststroke; 11, 200m breaststroke; 12, 100m butterfly; 13, 200m butterfly; 14, 50m freestyle; 15. 100m freestyle; 16, 200m freestyle; 17, 400m freestyle; 18, 800m freestyle; 19, 200m medley; 20, 400m medley; 21, 400m hurdles; 22, long jump; 23, high jump; 24, triple jump).

The correlation analysis results (Fig 3a) reveal a high positive correlation (*R*>0.7, *P*<0.05) among the TSRFs of all swimming events. Additionally, a high positive correlation (*R*>0.7, *P*<0.05) is observed between the 100m and 200m sprint events. Furthermore, there exists a high or moderate positive correlation between the marathon and all swimming events (*R* = 0.4 ~ 1.0, *P*<0.05). A moderate positive correlation is found between the 100m sprint and all swimming events (*R* = 0.4 ~ 0.7, *P*<0.05). Conversely, a moderate negative correlation is identified between the 3000m middle and long-distance running and all swimming events (*R* = -0.4 ~ -0.7, *P*<0.05). Notably, the network correlation analysis (Fig 3b) indicates a high positive correlation (*R*>0.7, *P*<0.05) and network connectivity between the TSRF of the annual world record in the marathon and all swimming events.

## Discussion

Predictive analyses of sports world records from 1992 to 2018 can be conducted using Gembris algorithmic model [1, 7]. The stochastic variation process of 400m and 3000m middle-distance running world records aligns well with the actual performance evolution, indicating a small approximation error and a superior fit of the prediction model for these two sports. This suggests no significant breakthrough in their performance over the 27 years, consistent with the generally low growth rate in track events in Result 3.1. Annual world records in sprinting, marathon, and swimming surpass predicted values, implying a substantial performance increase and systematic improvement from 1992 to 2018. This aligns with the notable growth rate change in sprinting, marathon, and swimming performance.

In the correlation analysis, a high positive correlation emerged among the TSRFs of all swimming events, suggesting no correlation between strokes (backstroke, breaststroke, butterfly, and freestyle) and event distances (sprints, intermediate, and long distances). This absence of a clear relationship allows for the emergence of "super athletes" like Michael Phelps (USA), Ian James Thorpe (Australia), and Sun Yang (China), who set new world records across various strokes and distances. Notably, the 100m and 200m sprints in track events exhibit a high positive correlation in TSRFs, exemplified by Jamaican sprinter Usain Bolt, holding world records in both (as of October 2023). Intriguingly, the marathon shows a similar strong positive correlation with all swimming events [8], and others, indicating a fractal relationship between time and events in top runners and swimmers’ athletic performance.

**Fig 4:**
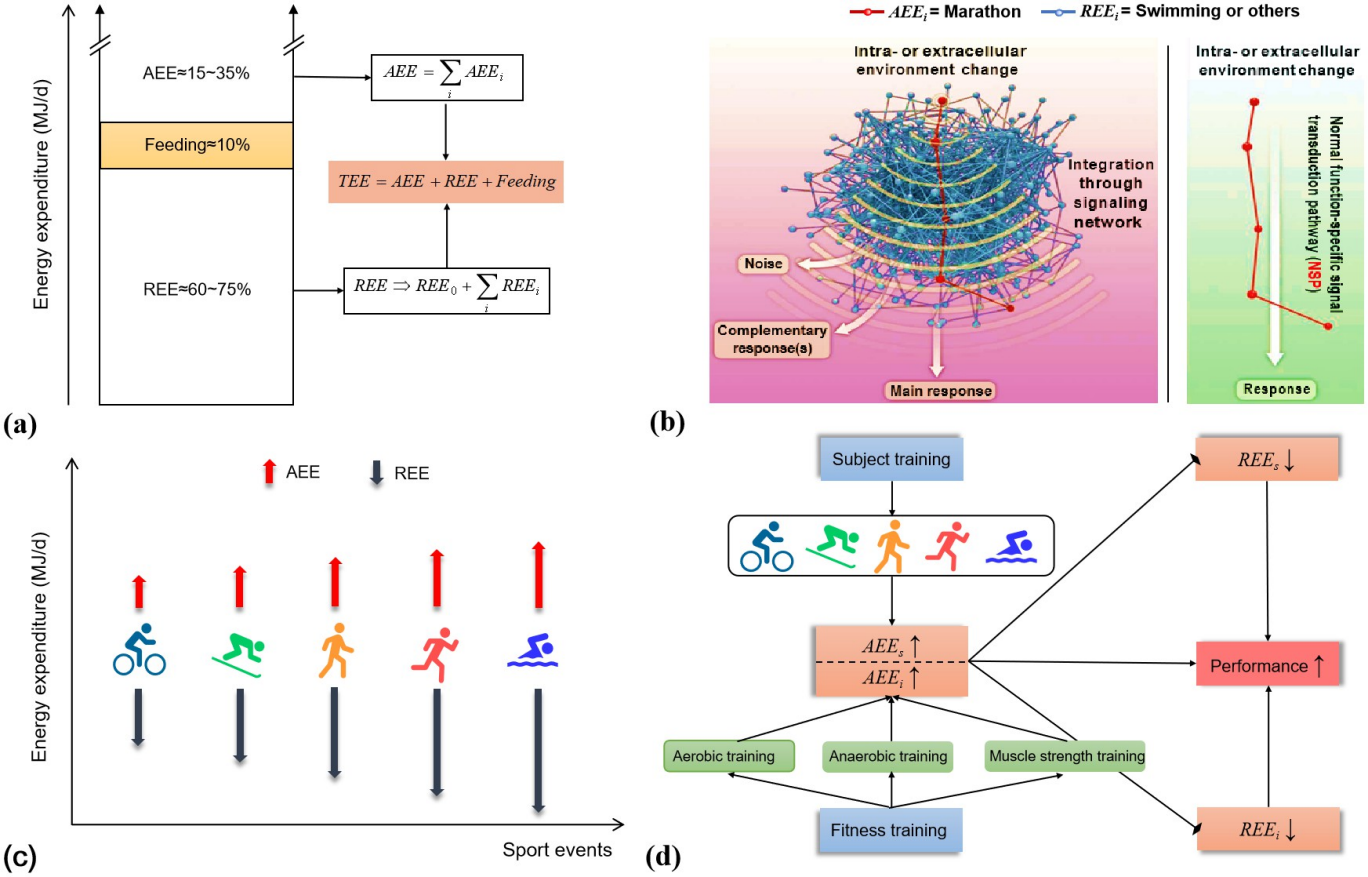
Correlation between sports events and energy expenditure. (a) The total daily energy expenditure (TEE) and its proportions of active energy expenditure (AEE), resting energy expenditure (REE) and Feeding, the sum of each type of active energy expenditure (*AEE*_*i*_) constitutes AEE, the sum of each type of resting energy expenditure (*REE*_*i*_) and the basal energy expenditure to maintain the body (*REE*_*0*_) constitutes REE. Thus, *TEE = AEE+ REE+ Feeding* [9]. (b) According to the normal function-specific signal transduction pathway (NSP) [16] diagram of energy expenditure of marathon adapted, the red line is the energy expenditure of marathon (*AEE*_*i*_) and the blue line is the resting energy expenditure of swimming or other sports (*REE*_*i*_). (c) AEE and REE diagrams of different sports events. (d) Subject training and fitness training improve the level of sports performance through the relationship between AEE and REE.

Sports records embody the human pursuit of optimal performance, a continual refinement of behavior with both explicit and implicit functions in each activity. Poehlman and Dvorak (9)utilizing the double-labelled water method to measure energy expenditure, revealed that activity energy expenditure (AEE) constitutes 15–35% of total energy expenditure (TEE) per day, contrasting with the dominant 60–75% of TEE attributed to resting energy expenditure (REE), leaving approximately 10% for feeding activity (Fig 4a). Enhancing the optimal exercise level primarily involves elevating AEE, markedly reducing the risk of all-cause mortality. A cohort study on over 2 million individuals found a positive association between higher AEE levels, increased activity skills, and reduced mortality risk [10]. Higher AEE levels were also linked to modulated rhythmic gene expression, manifesting as decreased body mass and increased maximal oxygen uptake [11]. Another avenue for intensifying activity is the reduction of REE. Studies indicate significant post-training reductions in REE after 40 weeks [12] and during 30 weeks of weight loss training [13]. Athletes commonly exhibit a lower resting heart rate, known as heart rate reserve or cardiac reserve function, contrasting with the general population. Therefore, the objective of exercise training may involve augmenting overall functional performance by increasing active energy expenditure and subsequently decreasing resting energy expenditure.

This paper marks the inaugural identification of a robust connection between the evolution of world records in the marathon and all swimming events. This seemingly counterintuitive revelation necessitates revisiting Aristotle’s primary principle of nature [14]. The foundational nature of exercise encompasses both external action and internal energy expenditure. Despite the marked distinctions in movement and energy expenditure between marathon and swimming, a profound interconnection emerges when scrutinized through the lens of activity and resting energy expenditure, as delineated in the preceding section. In elite marathon runners, the marathon assumes a paramount role in all activities. When engaged in marathon running, the associated activity energy expenditure (AEE) substantially rises, concurrently suppressing swimming’s resting energy expenditure (REE) linked with other sports. This phenomenon aligns with the normal function-specific signal transduction pathway (NSP) effect—a negative feedback mechanism. This mechanism ensures that the specific exercise’s function inhibits the activation of alternative signal transduction pathways in target tissue cells [15, 16]. Marathon-related target tissue cells, through cytokines or exosomes, activate the marathon NSP in a widespread manner. This strategically conserves energy expenditure, facilitating a more efficient marathon activity by suppressing swimming or analogous functions (Fig 4b).

In the realm of sports, variations in energy expenditure directly impact the interconnection between athletic specialties. Fig 4c illustrates the correlation between energy expenditure in different sports [17, 18]. When subjected to identical conditions, swimming events exhibit comparatively higher activity energy expenditure (AEE) levels due to the necessity of overcoming water resistance. Conversely, cycling or skating events demonstrate relatively lower AEE levels by utilizing tools to hasten movement. Swimming, on the other hand, boasts the lowest resting energy expenditure (REE) level, with running, walking, skating, and riding showcasing descending resting metabolic levels. Specialized training aims to elevate the AEE level of a specific event and diminish the non-specialized training REE level. Essentially, reducing REE levels in non-specific training compensates for the AEE levels in specific training. Therefore, alongside specialized training, reinforcing and supplementing non-specific training [4–6]. Furthermore, physical fitness represents the fundamental athletic prowess of the human body, encompassing strength, speed, endurance, coordination, flexibility, sensitivity, and other athletic qualities. It is a pivotal component of athletes’ competitive capabilities. Physical fitness training predominantly involves aerobic training, anaerobic training, and muscle strength training to enhance AEE levels across different abilities. Similarly, while augmenting the AEE level of a specific athletic ability, it concurrently diminishes the REE level of other athletic abilities. This approach ultimately serves the purpose of enhancing sports performance and athletic achievements (Fig 4d). In conclusion, alongside advancing the AEE level of specialization and physical abilities, it is imperative to consider the AEE level of non-specialized sports and diverse physical training. Ultimately, reducing the corresponding REE level broadens the scope of energy expenditure compensation, ensuring the full realization of sports performance.

The study acknowledges several limitations. Firstly, our results come from group data and do not provide a comprehensive evaluation of the long-term sports performance of specific athletes. Additionally, our model encounters challenges stemming from non-physiological factors such as environmental conditions, participation in the sport, and doping [19]. Particularly noteworthy is the trend of doping practices becoming more covert and sophisticated, which could potentially impact the progression of sports records in manners that current statistical models may fail to accurately capture [20]. Finally, The study’s primary focus on male sports records aimed to delve deeply into the evolution of sports records within specific gender groups. This targeted approach not only minimized the influence of confounding variables but also facilitated a more precise understanding of the mechanisms underlying male sports record evolution. However, acknowledging gender disparities is crucial for ensuring the comprehensive applicability of our findings. For future investigations, we advocate for similar in-depth analyses of female athletes’ sports records to enrich insight into gender-specific sports performance dynamics within specific contexts. Such comparative studies would contribute significantly to our understanding of how gender influences athletic performance under varying conditions. In addition, future research should include longitudinal studies in other sports or across longer periods, this would provide valuable insights for coaches to design more targeted training programs and interventions to optimize sports performance.

## Conclusion

This paper quantitatively analyzes the evolution of annual world records in 24 track and field and swimming events from 1992 to 2018. Specifically, swimming records have outpaced those in field and track events, with all field events showing negative growth. Correlation analyses reveal strong connections not only between swimming and sprint events but also between marathons and all swimming events. Additionally, the predictive model analysis confirms that these high correlations between events are non-random. Lastly, the paper explores the interaction pattern of the annual world records’ evolution from the perspective of energy expenditure. This perspective elevates active energy expenditure during high-quality performance in a specific sport (specialized or physical training). Simultaneously, it considers the active energy expenditure in non-specialized sports and diversified physical training, significantly reducing the corresponding resting energy expenditure. This broadens the space for energy expenditure compensation, providing an optimal physiological basis for full performance and enhancement.
