# Supplementary material for: Exploring the dynamics of sports records evolution through the gembris prediction model and network relevance analysis
Source: PLoS One. 2024 Sep 19;19(9):e0307796. doi: 10.1371/journal.pone.0307796 (PMC11412484; doi:10.1371/journal.pone.0307796)
Supplement: Attachment — Submitted filename: Response to Reviewers.doc [file pone.0307796.s001.doc]

**Dear Editor and Reviewer,**

·	Thanks very much for taking your time to review this manuscript. I really appreciate all your comments and suggestion! Please find our itemized responses in below and our revisions in the re-submitted files (Revised Manuscript with Track Changes). Thanks again!

**Editor #**

When submitting your revision, we need you to address these additional requirements.

1. Please ensure that your manuscript meets PLOS ONE's style requirements, including those for file naming. The PLOS ONE style templates can be found at 
https://journals.plos.org/plosone/s/file?id=wjVg/PLOSOne_formatting_sample_main_body.pdf and 
https://journals.plos.org/plosone/s/file?id=ba62/PLOSOne_formatting_sample_title_authors_affiliations.pdf

2. We note that the grant information you provided in the ‘Funding Information’ and ‘Financial Disclosure’ sections do not match.

When you resubmit, please ensure that you provide the correct grant numbers for the awards you received for your study in the ‘Funding Information’ section.

3. Thank you for stating the following financial disclosure: 
"This work was supported by Fundamental Research Funds for the Central University [No. PHD2023-02]; Aviation Sports Research Institute project of CAFUC [No. JG2022-34-02]."

Please state what role the funders took in the study.  If the funders had no role, please state: ""The funders had no role in study design, data collection and analysis, decision to publish, or preparation of the manuscript."" 
If this statement is not correct you must amend it as needed. 
Please include this amended Role of Funder statement in your cover letter; we will change the online submission form on your behalf.

4. Please provide a complete Data Availability Statement in the submission form, ensuring you include all necessary access information or a reason for why you are unable to make your data freely accessible. If your research concerns only data provided within your submission, please write "All data are in the manuscript and/or supporting information files" as your Data Availability Statement.

5. PLOS requires an ORCID iD for the corresponding author in Editorial Manager on papers submitted after December 6th, 2016. Please ensure that you have an ORCID iD and that it is validated in Editorial Manager. To do this, go to ‘Update my Information’ (in the upper left-hand corner of the main menu), and click on the Fetch/Validate link next to the ORCID field. This will take you to the ORCID site and allow you to create a new iD or authenticate a pre-existing iD in Editorial Manager. Please see the following video for instructions on linking an ORCID iD to your Editorial Manager account: https://www.youtube.com/watch?v=_xcclfuvtxQ

6. Please amend the manuscript submission data (via Edit Submission) to include author Dr. Mingliang Yang.

7. Please upload a copy of Figure 4, to which you refer in your text on page 5. If the figure is no longer to be included as part of the submission please remove all reference to it within the text.

8. Please review your reference list to ensure that it is complete and correct. If you have cited papers that have been retracted, please include the rationale for doing so in the manuscript text, or remove these references and replace them with relevant current references. Any changes to the reference list should be mentioned in the rebuttal letter that accompanies your revised manuscript. If you need to cite a retracted article, indicate the article’s retracted status in the References list and also include a citation and full reference for the retraction notice.

**The author's answer:**

1. Thank you for your comment! Our revised manuscript conform to PLOS ONE style requirements, including file naming requirements.
2. **Line 324-329**. We have re-examined the Grant Information and Financial Disclosures and confirmed the correct grant numbers: This research was funded by a Fundamental Research Funds for the Central University Awarded to LT with grant number PHD2023-02 and a Aviation Sports Research Institute project of Civil Aviation Flight University of China Awarded to ML with grant number 24CAFUC09034.
3. **Line 327-329**. We have added a statement of the role that funders play in research: The funders had no role in study design, data collection and analysis, decision to publish, or preparation of the manuscript.
4. **Line 330-334**. We added a data availability statement: The data that was used during this study was already publicly available. The sports records were obtained from from the International Amateur Athletic Federation (IAAF) (https://worldathletics.org/records/toplists) and the International Swimming Federation (Fédération Internationale de Natation Association, FINA) (https://www.worldaquatics.com/swimming/rankings).
5. **Line 1-2.** We added the ORCID iD of the first and corresponding authors:

Lu Tang, ID: https://orcid.org/0000-0002-8797-6318

Mingliang Yang, ID: https://orcid.org/0009-0009-1323-3763

6. We have updated the submission system data of corresponding author Mingliang Yang.
7. **Line 218-226.** Thank you very much for your constructive comments. We apologize for the mis-information in the last version. We have added **Figure 4** to the revised draft.
8. **Line 273-327.** We have referred to the PLOS ONE style of referencing as well as checking the relevant listings to ensure they are complete and correct.

**Reviewer 1#**

1、Consider analyzing or at least discussing why women's records were not included. This could provide a more holistic view of sports evolution.
2、Explicitly state any limitations of your methodology or data selection to provide context for your findings.
3、 Offering suggestions for future research, possibly including other sports or longitudinal studies spanning longer periods, could be valuable.

The manuscript is well-written, providing valuable insights into the evolution of sports records. The use of the Gembris prediction model and network relevance analysis is innovative and appropriately applied. With minor improvements, especially regarding the inclusion of limitations and potentially broadening the scope to include women's records, this paper could make a significant contribution to the field of sports science and physiology.

**The author's answer:**

1. **Line 233-241.** Thank you very much for your constructive comments! We clarified the reasons in our revision of the manuscript: The study's primary focus on male sports records aimed to delve deeply into the evolution of sports records within specific gender groups. This targeted approach not only minimized the influence of confounding variables but also facilitated a more precise understanding of the mechanisms underlying male sports record evolution. However, acknowledging gender disparities is crucial for ensuring the comprehensive applicability of our findings. For future investigations, we advocate for similar in-depth analyses of female athletes' sports records to enrich insight into gender-specific sports performance dynamics within specific contexts. Such comparative studies would contribute significantly to our understanding of how gender influences athletic performance under varying conditions.
2. **Line 227-243.** Thank you very much for your valuable comments, which we very much appreciate.  We already added limitations in the discussion section: The study acknowledges several limitations. Firstly, our results come from group data and do not provide a comprehensive evaluation of the long-term sports performance of specific athletes. Additionally, our model encounters challenges stemming from non-physiological factors such as environmental conditions, participation in the sport, and doping[1]. Particularly noteworthy is the trend of doping practices becoming more covert and sophisticated, which could potentially impact the progression of sports records in manners that current statistical models may fail to accurately capture[2]. Finally, The study's primary focus on male sports records aimed to delve deeply into the evolution of sports records within specific gender groups. This targeted approach not only minimized the influence of confounding variables but also facilitated a more precise understanding of the mechanisms underlying male sports record evolution. However, acknowledging gender disparities is crucial for ensuring the comprehensive applicability of our findings. For future investigations, we advocate for similar in-depth analyses of female athletes' sports records to enrich insight into gender-specific sport performance dynamics within specific contexts. Such comparative studies would contribute significantly to our understanding of how gender influences athletic performance under varying conditions. In addition, future research should include longitudinal studies in other sports or across longer periods, this would provide valuable insights for coaches to design more targeted training programs and interventions to optimize sports performance.

**3. Line 241-243.** We thank that reviewer for their keen eye. We have added your suggestions to the revised manuscript and given it relevance for sports training: In addition, future research should include longitudinal studies in other sports or across longer periods of time, this would provide valuable insights for coaches to design more targeted training programs and interventions to optimize sports performance.

**Reviewer 2#**

1. In the conclusion section, at the first line there is a minor correction. Where you have mentioned the duration of year from 1992 to 2008, Please correct it. Also the limitation and scope for further research can be included.

**The author's answer:**

1. **Line 247**. Thank you for pointing out such a cheap mistake, we are very sorry for our low-level mistakes, we have changed 2008 to 2018. And we already added limitations in the discussion section at **Line 227-243**: The study acknowledges several limitations. Firstly, our results come from group data and do not provide a comprehensive evaluation of the long-term sports performance of specific athletes. Additionally, our model encounters challenges stemming from non-physiological factors such as environmental conditions, participation in the sport, and doping[1]. Particularly noteworthy is the trend of doping practices becoming more covert and sophisticated, which could potentially impact the progression of sports records in manners that current statistical models may fail to accurately capture[2]. Finally, The study's primary focus on male sports records aimed to delve deeply into the evolution of sports records within specific gender groups. This targeted approach not only minimized the influence of confounding variables but also facilitated a more precise understanding of the mechanisms underlying male sports record evolution. However, acknowledging gender disparities is crucial for ensuring the comprehensive applicability of our findings. For future investigations, we advocate for similar in-depth analyses of female athletes' sports records to enrich insight into gender-specific sport performance dynamics within specific contexts. Such comparative studies would contribute significantly to our understanding of how gender influences athletic performance under varying conditions. In addition, future research should include longitudinal studies in other sports or across longer periods, this would provide valuable insights for coaches to design more targeted training programs and interventions to optimize sports performance.

**We sincerely appreciate your invaluable contributions to our paper. Your insightful comments and suggestions have been both encouraging and constructive, providing illumination for our future endeavors in this domain and offering valuable guidance for our ongoing research. We have addressed all the inquiries and meticulously revised our manuscript based on your thoughtful feedback. Should any further questions arise, please do not hesitate to contact us.**

**Once again, thank you for your time and attention, and we eagerly anticipate hearing from you soon!**
