# Supplementary material for: Exploring the dynamics of sports records evolution through the gembris prediction model and network relevance analysis
Source: PLoS One. 2024 Sep 19;19(9):e0307796. doi: 10.1371/journal.pone.0307796 (PMC11412484; doi:10.1371/journal.pone.0307796)
Supplement: Peer review history

# Peer review: Decision Letter 0

**DOI:** 10.1371/journal.pone.0307796.r001

**Authors:** Mukesh Kumar Sinha

12 Apr 2024

PONE-D-24-05017Exploring the Dynamics of Sports Records Evolution through the Gembris Prediction Model and Network Relevance AnalysisPLOS ONE

Dear Dr. Tang,

Thank you for submitting your manuscript to PLOS ONE. After careful consideration, we feel that it has merit but does not fully meet PLOS ONE’s publication criteria as it currently stands. Therefore, we invite you to submit a revised version of the manuscript that addresses the points raised during the review process.

Please submit your revised manuscript by May 27 2024 11:59PM. If you will need more time than this to complete your revisions, please reply to this message or contact the journal office at plosone@plos.org. When you're ready to submit your revision, log on to https://www.editorialmanager.com/pone/ and select the 'Submissions Needing Revision' folder to locate your manuscript file.

Please include the following items when submitting your revised manuscript:

- A rebuttal letter that responds to each point raised by the academic editor and reviewer(s). You should upload this letter as a separate file labeled 'Response to Reviewers'.
- A marked-up copy of your manuscript that highlights changes made to the original version. You should upload this as a separate file labeled 'Revised Manuscript with Track Changes'.
- An unmarked version of your revised paper without tracked changes. You should upload this as a separate file labeled 'Manuscript'.

If you would like to make changes to your financial disclosure, please include your updated statement in your cover letter. Guidelines for resubmitting your figure files are available below the reviewer comments at the end of this letter.

If applicable, we recommend that you deposit your laboratory protocols in protocols.io to enhance the reproducibility of your results. Protocols.io assigns your protocol its own identifier (DOI) so that it can be cited independently in the future. For instructions see: https://journals.plos.org/plosone/s/submission-guidelines#loc-laboratory-protocols. Additionally, PLOS ONE offers an option for publishing peer-reviewed Lab Protocol articles, which describe protocols hosted on protocols.io. Read more information on sharing protocols at https://plos.org/protocols?utm_medium=editorial-email&utm_source=authorletters&utm_campaign=protocols.

We look forward to receiving your revised manuscript.

Kind regards,

Mukesh Kumar Sinha

Academic Editor

PLOS ONE

Journal Requirements:

When submitting your revision, we need you to address these additional requirements.

1. Please ensure that your manuscript meets PLOS ONE's style requirements, including those for file naming. The PLOS ONE style templates can be found at 

https://journals.plos.org/plosone/s/file?id=wjVg/PLOSOne_formatting_sample_main_body.pdf and 

https://journals.plos.org/plosone/s/file?id=ba62/PLOSOne_formatting_sample_title_authors_affiliations.pdf

2. We note that the grant information you provided in the ‘Funding Information’ and ‘Financial Disclosure’ sections do not match. 

When you resubmit, please ensure that you provide the correct grant numbers for the awards you received for your study in the ‘Funding Information’ section.

3. Thank you for stating the following financial disclosure: 

"This work was supported by Fundamental Research Funds for the Central University [No. PHD2023-02]; Aviation Sports Research Institute project of CAFUC [No. JG2022-34-02]."

Please state what role the funders took in the study.  If the funders had no role, please state: ""The funders had no role in study design, data collection and analysis, decision to publish, or preparation of the manuscript."" 

If this statement is not correct you must amend it as needed. 

Please include this amended Role of Funder statement in your cover letter; we will change the online submission form on your behalf.

4. Please provide a complete Data Availability Statement in the submission form, ensuring you include all necessary access information or a reason for why you are unable to make your data freely accessible. If your research concerns only data provided within your submission, please write "All data are in the manuscript and/or supporting information files" as your Data Availability Statement.

5. PLOS requires an ORCID iD for the corresponding author in Editorial Manager on papers submitted after December 6th, 2016. Please ensure that you have an ORCID iD and that it is validated in Editorial Manager. To do this, go to ‘Update my Information’ (in the upper left-hand corner of the main menu), and click on the Fetch/Validate link next to the ORCID field. This will take you to the ORCID site and allow you to create a new iD or authenticate a pre-existing iD in Editorial Manager. Please see the following video for instructions on linking an ORCID iD to your Editorial Manager account: https://www.youtube.com/watch?v=_xcclfuvtxQ

6. Please amend the manuscript submission data (via Edit Submission) to include author Dr. Mingliang Yang.

7. Please upload a copy of Figure 4, to which you refer in your text on page 5. If the figure is no longer to be included as part of the submission please remove all reference to it within the text.

8. Please review your reference list to ensure that it is complete and correct. If you have cited papers that have been retracted, please include the rationale for doing so in the manuscript text, or remove these references and replace them with relevant current references. Any changes to the reference list should be mentioned in the rebuttal letter that accompanies your revised manuscript. If you need to cite a retracted article, indicate the article’s retracted status in the References list and also include a citation and full reference for the retraction notice.

[Note: HTML markup is below. Please do not edit.]

Reviewers' comments:

Reviewer's Responses to Questions

**Comments to the Author**

1. Is the manuscript technically sound, and do the data support the conclusions?

The manuscript must describe a technically sound piece of scientific research with data that supports the conclusions. Experiments must have been conducted rigorously, with appropriate controls, replication, and sample sizes. The conclusions must be drawn appropriately based on the data presented. 

Reviewer #1: Yes

Reviewer #2: Yes

**********

2. Has the statistical analysis been performed appropriately and rigorously? 

Reviewer #1: Yes

Reviewer #2: Yes

**********

3. Have the authors made all data underlying the findings in their manuscript fully available?

The PLOS Data policy requires authors to make all data underlying the findings described in their manuscript fully available without restriction, with rare exception (please refer to the Data Availability Statement in the manuscript PDF file). The data should be provided as part of the manuscript or its supporting information, or deposited to a public repository. For example, in addition to summary statistics, the data points behind means, medians and variance measures should be available. If there are restrictions on publicly sharing data—e.g. participant privacy or use of data from a third party—those must be specified.

Reviewer #1: Yes

Reviewer #2: Yes

**********

4. Is the manuscript presented in an intelligible fashion and written in standard English?

PLOS ONE does not copyedit accepted manuscripts, so the language in submitted articles must be clear, correct, and unambiguous. Any typographical or grammatical errors should be corrected at revision, so please note any specific errors here.

Reviewer #1: Yes

Reviewer #2: Yes

**********

5. Review Comments to the Author

Please use the space provided to explain your answers to the questions above. You may also include additional comments for the author, including concerns about dual publication, research ethics, or publication ethics. (Please upload your review as an attachment if it exceeds 20,000 characters)

Reviewer #1: 1、Consider analyzing or at least discussing why women's records were not included. This could provide a more holistic view of sports evolution.

2、Explicitly state any limitations of your methodology or data selection to provide context for your findings.

3、 Offering suggestions for future research, possibly including other sports or longitudinal studies spanning longer periods, could be valuable.

The manuscript is well-written, providing valuable insights into the evolution of sports records. The use of the Gembris prediction model and network relevance analysis is innovative and appropriately applied. With minor improvements, especially regarding the inclusion of limitations and potentially broadening the scope to include women's records, this paper could make a significant contribution to the field of sports science and physiology.

Reviewer #2: In the conclusion section, at the first line there is a minor correction. Where you have mentioned the duration of year from 1992 to 2008, Please correct it. Also the limitation and scope for further research can be included.

**********

6. PLOS authors have the option to publish the peer review history of their article (what does this mean?). If published, this will include your full peer review and any attached files.

If you choose “no”, your identity will remain anonymous but your review may still be made public.

**Do you want your identity to be public for this peer review?** For information about this choice, including consent withdrawal, please see our Privacy Policy.

Reviewer #1: No

Reviewer #2: No

**********

[NOTE: If reviewer comments were submitted as an attachment file, they will be attached to this email and accessible via the submission site. Please log into your account, locate the manuscript record, and check for the action link "View Attachments". If this link does not appear, there are no attachment files.]

While revising your submission, please upload your figure files to the Preflight Analysis and Conversion Engine (PACE) digital diagnostic tool, https://pacev2.apexcovantage.com/. PACE helps ensure that figures meet PLOS requirements. To use PACE, you must first register as a user. Registration is free. Then, login and navigate to the UPLOAD tab, where you will find detailed instructions on how to use the tool. If you encounter any issues or have any questions when using PACE, please email PLOS at figures@plos.org. Please note that Supporting Information files do not need this step.

# Author comment: Author response to Decision Letter 0

**DOI:** 10.1371/journal.pone.0307796.r002

19 Jun 2024

Dear Editor and Reviewer, 

· Thanks very much for taking your time to review this manuscript. I really appreciate all your comments and suggestion! Please find our itemized responses in below and our revisions in the re-submitted files (Revised Manuscript with Track Changes). Thanks again! 

Editor #

When submitting your revision, we need you to address these additional requirements.

1. Please ensure that your manuscript meets PLOS ONE's style requirements, including those for file naming. The PLOS ONE style templates can be found at 

https://journals.plos.org/plosone/s/file?id=wjVg/PLOSOne_formatting_sample_main_body.pdf and 

https://journals.plos.org/plosone/s/file?id=ba62/PLOSOne_formatting_sample_title_authors_affiliations.pdf

2. We note that the grant information you provided in the ‘Funding Information’ and ‘Financial Disclosure’ sections do not match. 

When you resubmit, please ensure that you provide the correct grant numbers for the awards you received for your study in the ‘Funding Information’ section.

3. Thank you for stating the following financial disclosure: 

"This work was supported by Fundamental Research Funds for the Central University [No. PHD2023-02]; Aviation Sports Research Institute project of CAFUC [No. JG2022-34-02]."

Please state what role the funders took in the study.  If the funders had no role, please state: ""The funders had no role in study design, data collection and analysis, decision to publish, or preparation of the manuscript."" 

If this statement is not correct you must amend it as needed. 

Please include this amended Role of Funder statement in your cover letter; we will change the online submission form on your behalf.

4. Please provide a complete Data Availability Statement in the submission form, ensuring you include all necessary access information or a reason for why you are unable to make your data freely accessible. If your research concerns only data provided within your submission, please write "All data are in the manuscript and/or supporting information files" as your Data Availability Statement.

5. PLOS requires an ORCID iD for the corresponding author in Editorial Manager on papers submitted after December 6th, 2016. Please ensure that you have an ORCID iD and that it is validated in Editorial Manager. To do this, go to ‘Update my Information’ (in the upper left-hand corner of the main menu), and click on the Fetch/Validate link next to the ORCID field. This will take you to the ORCID site and allow you to create a new iD or authenticate a pre-existing iD in Editorial Manager. Please see the following video for instructions on linking an ORCID iD to your Editorial Manager account: https://www.youtube.com/watch?v=_xcclfuvtxQ

6. Please amend the manuscript submission data (via Edit Submission) to include author Dr. Mingliang Yang.

7. Please upload a copy of Figure 4, to which you refer in your text on page 5. If the figure is no longer to be included as part of the submission please remove all reference to it within the text.

8. Please review your reference list to ensure that it is complete and correct. If you have cited papers that have been retracted, please include the rationale for doing so in the manuscript text, or remove these references and replace them with relevant current references. Any changes to the reference list should be mentioned in the rebuttal letter that accompanies your revised manuscript. If you need to cite a retracted article, indicate the article’s retracted status in the References list and also include a citation and full reference for the retraction notice.

The author's answer: 

1.Thank you for your comment! Our revised manuscript conform to PLOS ONE style requirements, including file naming requirements.

2.Line 323-328. We have re-examined the Grant Information and Financial Disclosures and confirmed the correct grant numbers: This research was funded by a Fundamental Research Funds for the Central University Awarded to LT with grant number PHD2023-02 and a Aviation Sports Research Institute project of Civil Aviation Flight University of China Awarded to ML with grant number 24CAFUC09034.

3.Line 326-328. We have added a statement of the role that funders play in research: The funders had no role in study design, data collection and analysis, decision to publish, or preparation of the manuscript.

4.Line 341-345. We added a data availability statement: The data that was used during this study was already publicly available. The sports records were obtained from from the International Amateur Athletic Federation (IAAF) (www.worldathletics.org) and the International Swimming Federation (Fédération Internationale de Natation Association, FINA) (www.fina.org).

5.Line 1-2. We added the ORCID iD of the first and corresponding authors: 

Lu Tang, ID: https://orcid.org/0000-0002-8797-6318

Mingliang Yang, ID: https://orcid.org/0009-0009-1323-3763

6.We have updated the submission system data of corresponding author Mingliang Yang.

7.Line 217-226. Thank you very much for your constructive comments. We apologize for the mis-information in the last version. We have added Figure 4 to the revised draft.

8.Line 273-327. We have referred to the PLOS ONE style of referencing as well as checking the relevant listings to ensure they are complete and correct.

Reviewer 1#

1、Consider analyzing or at least discussing why women's records were not included. This could provide a more holistic view of sports evolution.

2、Explicitly state any limitations of your methodology or data selection to provide context for your findings.

3、 Offering suggestions for future research, possibly including other sports or longitudinal studies spanning longer periods, could be valuable.

The manuscript is well-written, providing valuable insights into the evolution of sports records. The use of the Gembris prediction model and network relevance analysis is innovative and appropriately applied. With minor improvements, especially regarding the inclusion of limitations and potentially broadening the scope to include women's records, this paper could make a significant contribution to the field of sports science and physiology.

The author's answer: 

1.Line 233-241. Thank you very much for your constructive comments! We clarified the reasons in our revision of the manuscript: The study's primary focus on male sports records aimed to delve deeply into the evolution of sports records within specific gender groups. This targeted approach not only minimized the influence of confounding variables but also facilitated a more precise understanding of the mechanisms underlying male sports record evolution. However, acknowledging gender disparities is crucial for ensuring the comprehensive applicability of our findings. For future investigations, we advocate for similar in-depth analyses of female athletes' sports records to enrich insight into gender-specific sports performance dynamics within specific contexts. Such comparative studies would contribute significantly to our understanding of how gender influences athletic performance under varying conditions. 

2.Line 227-243. Thank you very much for your valuable comments, which we very much appreciate.  We already added limitations in the discussion section: The study acknowledges several limitations. Firstly, our results come from group data and do not provide a comprehensive evaluation of the long-term sports performance of specific athletes. Additionally, our model encounters challenges stemming from non-physiological factors such as environmental conditions, participation in the sport, and doping[1]. Particularly noteworthy is the trend of doping practices becoming more covert and sophisticated, which could potentially impact the progression of sports records in manners that current statistical models may fail to accurately capture[2]. Finally, The study's primary focus on male sports records aimed to delve deeply into the evolution of sports records within specific gender groups. This targeted approach not only minimized the influence of confounding variables but also facilitated a more precise understanding of the mechanisms underlying male sports record evolution. However, acknowledging gender disparities is crucial for ensuring the comprehensive applicability of our findings. For future investigations, we advocate for similar in-depth analyses of female athletes' sports records to enrich insight into gender-specific sport performance dynamics within specific contexts. Such comparative studies would contribute significantly to our understanding of how gender influences athletic performance under varying conditions. In addition, future research should include longitudinal studies in other sports or across longer periods, this would provide valuable insights for coaches to design more targeted training programs and interventions to optimize sports performance.

1. Tang L, Ding W, Liu C. Scaling Invariance of Sports Sex Gap. Front Physiol. 2020;11(1654):606769. doi: https://doi.org/10.3389/fphys.2020.606769. PubMed Central PMCID: PMC33362581.

2. Giuseppe L, Giuseppe B, Favaloro EJ, Joern R, Nicola M. Updates on improvement of human athletic performance: focus on world records in athletics. Br Med Bull. 2008;87(1):7-15. doi: https://doi.org/10.1093/bmb/ldn029. PubMed Central PMCID: PMC18723588.

3. Line 241-243. We thank that reviewer for their keen eye. We have added your suggestions to the revised manuscript and given it relevance for sports training: In addition, future research should include longitudinal studies in other sports or across longer periods of time, this would provide valuable insights for coaches to design more targeted training programs and interventions to optimize sports performance.

Reviewer 2#

1. In the conclusion section, at the first line there is a minor correction. Where you have mentioned the duration of year from 1992 to 2008, Please correct it. Also the limitation and scope for further research can be included.

The author's answer: 

1.Line 247. Thank you for pointing out such a cheap mistake, we are very sorry for our low-level mistakes, we have changed 2008 to 2018. And we already added limitations in the discussion section at Line 227-243: The study acknowledges several limitations. Firstly, our results come from group data and do not provide a comprehensive evaluation of the long-term sports performance of specific athletes. Additionally, our model encounters challenges stemming from non-physiological factors such as environmental conditions, participation in the sport, and doping[1]. Particularly noteworthy is the trend of doping practices becoming more covert and sophisticated, which could potentially impact the progression of sports records in manners that current statistical models may fail to accurately capture[2]. Finally, The study's primary focus on male sports records aimed to delve deeply into the evolution of sports records within specific gender groups. This targeted approach not only minimized the influence of confounding variables but also facilitated a more precise understanding of the mechanisms underlying male sports record evolution. However, acknowledging gender disparities is crucial for ensuring the comprehensive applicability of our findings. For future investigations, we advocate for similar in-depth analyses of female athletes' sports records to enrich insight into gender-specific sport performance dynamics within specific contexts. Such comparative studies would contribute significantly to our understanding of how gender influences athletic performance under varying conditions. In addition, future research should include longitudinal studies in other sports or across longer periods, this would provide valuable insights for coaches to design more targeted training programs and interventions to optimize sports performance.

1. Tang L, Ding W, Liu C. Scaling Invariance of Sports Sex Gap. Front Physiol. 2020;11(1654):606769. doi: https://doi.org/10.3389/fphys.2020.606769. PubMed Central PMCID: PMC33362581.

2. Giuseppe L, Giuseppe B, Favaloro EJ, Joern R, Nicola M. Updates on improvement of human athletic performance: focus on world records in athletics. Br Med Bull. 2008;87(1):7-15. doi: https://doi.org/10.1093/bmb/ldn029. PubMed Central PMCID: PMC18723588.

We sincerely appreciate your invaluable contributions to our paper. Your insightful comments and suggestions have been both encouraging and constructive, providing illumination for our future endeavors in this domain and offering valuable guidance for our ongoing research. We have addressed all the inquiries and meticulously revised our manuscript based on your thoughtful feedback. Should any further questions arise, please do not hesitate to contact us. 

Once again, thank you for your time and attention, and we eagerly anticipate hearing from you soon!

Attachment Submitted filename: Response to Reviewers.doc

# Editor report: Decision Letter 1

**DOI:** 10.1371/journal.pone.0307796.r003

**Authors:** Mukesh Kumar Sinha

11 Jul 2024

Exploring the Dynamics of Sports Records Evolution through the Gembris Prediction Model and Network Relevance Analysis

PONE-D-24-05017R1

Dear Dr. Tang,

We’re pleased to inform you that your manuscript has been judged scientifically suitable for publication and will be formally accepted for publication once it meets all outstanding technical requirements.

Within one week, you’ll receive an e-mail detailing the required amendments. When these have been addressed, you’ll receive a formal acceptance letter and your manuscript will be scheduled for publication.

An invoice will be generated when your article is formally accepted. Please note, if your institution has a publishing partnership with PLOS and your article meets the relevant criteria, all or part of your publication costs will be covered. Please make sure your user information is up-to-date by logging into Editorial Manager at Editorial Manager® and clicking the ‘Update My Information' link at the top of the page. If you have any questions relating to publication charges, please contact our Author Billing department directly at authorbilling@plos.org.

If your institution or institutions have a press office, please notify them about your upcoming paper to help maximize its impact. If they’ll be preparing press materials, please inform our press team as soon as possible -- no later than 48 hours after receiving the formal acceptance. Your manuscript will remain under strict press embargo until 2 pm Eastern Time on the date of publication. For more information, please contact onepress@plos.org.

Kind regards,

Mukesh Kumar Sinha

Academic Editor

PLOS ONE

Additional Editor Comments (optional):

Reviewers' comments:

# Editor report: Acceptance letter

**DOI:** 10.1371/journal.pone.0307796.r004

**Authors:** Mukesh Kumar Sinha

10 Sep 2024

PONE-D-24-05017R1 

PLOS ONE

Dear Dr. Tang, 

I'm pleased to inform you that your manuscript has been deemed suitable for publication in PLOS ONE. Congratulations! Your manuscript is now being handed over to our production team.

At this stage, our production department will prepare your paper for publication. This includes ensuring the following:

* All references, tables, and figures are properly cited

* All relevant supporting information is included in the manuscript submission,

* There are no issues that prevent the paper from being properly typeset

If revisions are needed, the production department will contact you directly to resolve them. If no revisions are needed, you will receive an email when the publication date has been set. At this time, we do not offer pre-publication proofs to authors during production of the accepted work. Please keep in mind that we are working through a large volume of accepted articles, so please give us a few weeks to review your paper and let you know the next and final steps. 

Lastly, if your institution or institutions have a press office, please let them know about your upcoming paper now to help maximize its impact. If they'll be preparing press materials, please inform our press team within the next 48 hours. Your manuscript will remain under strict press embargo until 2 pm Eastern Time on the date of publication. For more information, please contact onepress@plos.org.

If we can help with anything else, please email us at customercare@plos.org.

Thank you for submitting your work to PLOS ONE and supporting open access. 

Kind regards, 

PLOS ONE Editorial Office Staff

on behalf of

Dr. Mukesh Kumar Sinha 

Academic Editor

PLOS ONE
